# Expression of Matrix Metalloproteinases 7 and 9, Desmin, Alpha-Smooth Muscle Actin and Caldesmon, in Odontogenic Keratocyst Associated with NBCCS, Recurrent and Sporadic Keratocysts

**DOI:** 10.3390/biom12060775

**Published:** 2022-06-02

**Authors:** Carla Loreto, Alessandro Polizzi, Veronica Filetti, Giuseppe Pannone, Jean Nunes Dos Santos, Pietro Venezia, Rosalia Leonardi, Gaetano Isola

**Affiliations:** 1 Human Anatomy and Histology, Department of Biomedical and Biotechnology Sciences, University of Catania, 95123 Catania, Italy; cloreto@unict.it (C.L.); verofiletti@gmail.com (V.F.); 2 Department of Medical and Surgical Sciences, University of Catania, 95123 Catania, Italy; pierovenezia@gmail.com (P.V.); rleonard@unict.it (R.L.); gaetano.isola@unict.it (G.I.); 3 Anatomic Pathology Unit, Department of Clinic and Experimental Medicine, University of Foggia, 71122 Foggia, Italy; giuseppe.pannone@unifg.it; 4 School of Dentistry, Federal University of Bahia, Salvador 40000-000, Brazil; jeanpatol@gmail.com

**Keywords:** immunohistochemistry, NBCCS, OKCs, myofibroblasts, MMP-7, MMP-9, desmin, alpha-SMA, caldesmon

## Abstract

Nevoid basal cell carcinoma syndrome (NBCCS) associated odontogenic keratocysts (OKCs) show more aggressive behavior and it has a higher frequency of relapse than non-syndromic OKCs. Stromal myofibroblasts (MFs), characterized by α-smooth muscle actin (αSMA), desmin and caldesmon expression, and metalloproteinases (MMPs) have an essential role in the remodeling of the extracellular matrix (ECM). The aim of the study is to analyze the immunohistochemical expression of MMP-7, MMP-9, αSMA and other new markers in the study of OKCs MFs such as desmin and caldesmon in NBCCS-associated OKCs compared to recurrent and sporadic keratocysts. Fourty 40 patients (23 M and 17 F) underwent surgery to remove the OKCs. The histological sections in paraffin were incubated with markers antibodies and a semi-quantitative score was used to evaluate the immunoreactivity. Densitometric analysis showed a very significantly increased expression of αSMA, caldesmon, MMP-7 and MMP-9 in NBCCS-OKCs compared to non-syndromic OKCs (*p* < 0.001). However, desmin showed a not significant increased expression in non-syndromic OKC compared to NBCCS-OKCs specimens in which desmin was slightly or not at all expressed. NBCSS-OKCs showed a greater distribution of MFs compared to the other OKCs subtypes. Further studies will be needed to evaluate whether the different expressions of these markers can be correlated to a different clinical behavior.

## 1. Introduction

Odontogenic keratocysts (OKCs) are osteolytic lesions localized in the jaws that can be sporadic or associated with nevoid basal cell carcinoma syndrome (NBCCS) [1]. Based on the evidence available to date, the World Health Organization (WHO) consensus group recently concluded that there is not sufficient data to support the neoplastic origin of OKCs, therefore the term “keratocystic odontogenic tumour” (KCOT) is no longer contemplated in the 4th edition of the WHO Classification of Head and Neck tumors [2]. However, OKCs show a more local aggressive behavior and higher risk of recurrence than other odontogenic cysts [3].

Furthermore, NBCCS-OKCs show faster growth, more aggressive tissue infiltration, and a higher recurrence rate than non-syndromic OKCs [4]. NBCCS-OKCs usually appear as multiple lesions of the jaws, which cause wide bone resorption, increasing the risk of fractures [5].

The participation of metalloproteinases (MMPs, as MMP-1, MMP-7, MMP-9, MMP-13, and MMP-26) in the biological behavior and invasion mechanisms of OKCs lesions has been demonstrated in many articles [6,7,8,9,10]. They can modify the structural and functional components of the extracellular matrix (ECM). For example, an investigation showed that the overexpression of MMP-13 in the stromal tissue might explain the NBCCS-OKCs higher aggressiveness than recurrent and sporadic keratocysts [6]. Furthermore, the elevated MMP-1 concentration in the parenchyma of OKCs is correlated with an increased number of myofibroblasts (MFs) [4], which are specialized fibroblasts with features of smooth muscle differentiation. Studies suggested that MMP-9 is involved in the expansion of odontogenic cysts and it has been demonstrated that its higher expression in OKCs could explain the more aggressive biologic behavior compared to dentigerous and radicular cysts [7]. A more recent study revealed that MMP-9 was more expressed in dentigerous cysts, OKCs and ameloblastomas compared to dental follicles. However, no differentiation was made between the various types of OKCs. Regarding MMP-7 (involved in the degradation of the basement membrane, cell proliferation, apoptosis, invasion and metastases [11]) a single study did not show a significant difference in epithelial immunoreactivity between NBCCS-OKCs and non-syndromic OKCs [10]. Further studies are needed to validate the role of these MMPs in the various types of OKCs.

The OKCs growth mechanisms are not fully clarified; however, evidence have shown the involvement of the parenchymal proliferative activity and its relationship to the stroma [12]. Stromal MF is often characterized by α-smooth muscle actin (αSMA), desmin and caldesmon expression and it has an essential role in the remodeling of the ECM and in the promotion of cancer cell invasion [13]. Many authors have highlighted the role of the stroma in the aggressiveness of many cancers such as esophageal, gastric, lung and oral carcinoma and also in oral potentially malignant lesions such as oral leukoplakia and oral submucous fibrosis [14]. Malignant cells of scirrhous gastric carcinoma, through the production of TGF-β, increase the number of cancer-associated MFs, promoting tumor growth and metastasis [15]. It was shown that TGF-β produced from esophageal squamous cell carcinoma (ESCC) promotes MFs VEGF production and consequently angiogenesis [16].

Furthermore, MFs are localized in the stroma of oral squamous cell carcinomas (OSCC) and may promote tumor proliferation and invasion through the secretion of activin A [17]. The distribution of MF, evaluated using αSMA, was not detected in healthy oral mucosa but in oral lesions such as oral leukoplakia, oral submucous fibrosis and OSCC [18]. This may be explained because epithelial cancers often show changes in the connective tissue called “stromal reaction”. One example of a stromal reaction is the differentiation of fibroblasts to MF. These MF produce growth factors and cytokines, resulting in a stimulation of epithelial cell proliferation, neoangiogenesis, basement membrane disruption, invasion and metastasis [19]. Although histologically identical, NBCCS-OKCs, compared to OKCs, may show parakeratinization, intramural epithelial remnants, and satellite cysts more frequently. Furthermore, it was demonstrated that NBCCS-associated OKC myofibroblasts show higher proliferative and osteoclastogenic capacity than non-syndromic OKC [5].

The aim of this investigation is to describe the immunohistochemical expression of MMP-7, MMP-9, αSMA and other new markers in the study of OKCs MFs such as desmin and caldesmon in NBCCS-OKCs compared to recurrent and sporadic keratocysts.

## 2. Materials and Methods

### 2.1. Patients Selected for the Study

After the formal approval from the Institutional Ethics Committee of the University of Catania, Italy (121/20/PO, 28 September 20), 40 cases of OKCs (23 M and 17 F, mean age 32 ± 8.7 years) were retrieved from the archives of the Oral Pathology Unit. All the OKCs lesions (4 in the maxilla and 36 in the mandible) were surgically removed through the same Partsch II approach. The classification of the lesions was carried out following precise criteria:

- Sporadic OKCs (sp-OKCs, *n* = 19) were primary lesions never cured;
- Recurrent OKCs (sr-OKCs, *n* = 9) in case of recurrence > 1 year after surgical removal;
- NBCCS-OKCs (NBCCS-OKCs, *n* = 12) when the criteria for NBCCS diagnosis were fulfilled [20]. Furthermore, PTCH gene mutations have been confirmed through molecular analysis, as previously described [21].

The parameters of the WHO classification of head and neck tumors [22] were considered when comparing clinical, radiological, and histological available data in order to make diagnoses. Non-inflamed follicular dentigerous cysts (DC, *n* = 7) were used as controls, since they are cystic lesions easily available and with a normally less aggressive behavior compared to OCKs. Subjects with these characteristics were excluded from the study: <18 years, pregnancy, systemic diseases, habitual use of anti-inflammatory drugs and chemotherapy.

### 2.2. Histological Analysis

All samples were washed in Phosphate Buffered Saline (PBS; Sigma, Milan, Italy) and subsequently were fixed in 10% buffered formalin for 2 h. The dehydration and clarification process were carried out the following day using graduated ethanol and xylene. Finally, the tissues were paraffin-embedded without altering their anatomical orientation, as previously described [23]. Using a microtome, 4–5 μm thick sections were obtained, which were maintained at room temperature after being mounted on silane-coated slides (Dako, Glostrup, Denmark). All sections were observed for general morphological analysis with a Zeiss Axioplan light microscope (Carl Zeiss, Oberkochen, Germany) using Hematoxylin and Eosin (H&E) staining. Best representative photomicrographs were captured using a Zeiss AxioCam MRc5 digital camera (Carl Zeiss). Two independent pathologists have analyzed the slides.

### 2.3. Immunohistochemical Analysis

After an overnight wash in PBS, all samples were fixed in 10% buffered formalin for 2 h, dehydrated using graduated ethanol and embedded in paraffin. Using a microtome, 3–4 μm thick sections were cut, mounted on silane-coated slides and air-dried. The immunohistochemical analysis was performed as previously described [24]. After being dewaxed in xylene and rehydrated with graded ethanol, the sections were incubated in 0.3% H_2_O_2_/methanol solution for 30 min (to neutralize endogenous peroxidases) and finally rinsed in PBS for 20 min. Antigen was retrieved in capped polypropylene slide-holders with citrate buffer (10 mM citric acid, 0.05% Tween 20, pH 6.0; Bio-Optica, Milan, Italy) using a 750 Watt microwave oven (3 × 5 min). All the sections were incubated at 4 °C overnight with the following primary antibodies: mouse monoclonal anti-αSMA (clone 1A4-Dako Corporation, Carpinteria, CA, USA) diluted 1:150; mouse monoclonal anti-MMP-7 (sc-80205-Santa Cruz Biotechnology, Inc., Dallas, TX, USA) diluted 1:100; mouse monoclonal anti-MMP-9 (sc-21733MMP-Santa Cruz Biotechnology, Inc., Dallas, TX, USA) diluted 1:100; rabbit monoclonal anti-desmin (SP138-Abcam, Cambridge, UK) diluted 1:100; mouse monoclonal anti-caldesmon (ab233987-Abcam, Cambridge, UK) diluted 1:150. All dilutions were performed in PBS. The generated immune complexes were treated and bound to biotinylated antibodies for 10 min at room temperature and, were finally detected using peroxidase-labeled streptavidin (LSAB + System-HRP, K0690; Dako), incubated for 10 min at room temperature. As previously described [25], 3,3-diaminobenzidine/0.02% H_2_O_2_ solution (DAB substrate Chromogen System; Dako) was used to visualize the immunoreaction. A lightly counterstaining of the sections was carried out with Mayer’s hematoxylin (Histolab Products AB, Göteborg, Sweden) mounting them in GVA (Zymed Laboratories, San Francisco, CA, USA). The observation and photomicrographs were realized as described above.

Immunoreactivity was considered as positive if brown chromogen was observed [26]. Colon adenocarcinoma harboring beta-catenin mutation served as a positive control, whereas negative controls were realized through PBS-treated sections without the primary antibodies. Morphometric and densitometric analysis were performed through a random selection of 7 field (≈600.000 μm^2^) from each section. Regarding the morphometric analysis, the % positivity of dark brown pixels of the analyzed fields expressed the percentage areas stained with αSMA, MMP-7, MMP-9, desmin, and caldesmon antibodies. Concerning the densitometric analysis, the densitometric count (pixel2) of the positive analyzed fields’, dark brown pixels expressed the levels (high/low) of staining intensity of positive areas. MFs were detected though brown staining exhibition. The sum of the values obtained for each field and the total number of anti-α-SMA-positive cells were calculated. The mean density of anti-α-SMA positive cells per mm^2^ was calculated starting from the total number of anti-α-SMA-positive cells. These parameters were calculated using software for image acquisition (AxioVision Release 4.8.2-SP2 Software, Carl Zeiss Microscopy GmbH, Jena, Germany) and were expressed as mean ± standard deviation (SD). Photomicrographs were obtained as described above.

### 2.4. Statistical Analysis

The data were plotted using Prism for Windows v 7.00 (Graphpad Software; San Diego, CA, USA). The data normality was tested using the Kolmogorov-Smirnov test that showed a normal distribution of the variables. The multiple *t*-test was used for comparisons between NBCCS-OKCs data and solitary OKCs data. *p*-values < 0.05 were considered statistically significant.

## 3. Results

### 3.1. MMP-7 Expression

**Figure 1:**
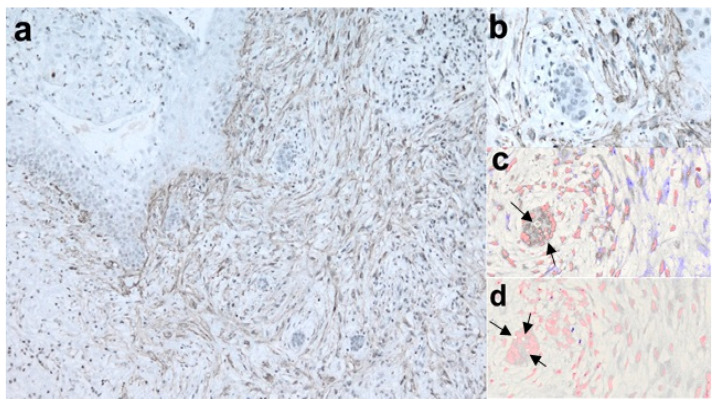
Interaction between epithelial cells and myofibroblasts during connective wall invasion in NBCCS-OKC. (**a**), αSMA staining depicts myofibroblasts surrounding invading epithelial droplets (LSAB-HRP, ×10; nuclear counterstaining with hematoxylin); (**b**), further magnification of a (LSAB-HRP, ×40); invading droplets have been further examined on serial sections by digital pathology showing high expression of MMP-7; (**c**), digital pathology, ×40, MMP-7 in violet and nuclear staining in red pseudocolors); (**d**), digital pathology ×40, MMP-9 in violet and nuclear staining in red pseudocolors), whereas MMP-9 showed a low level of expression [arrows indicated area of epithelial nuclei].

**Figure 2:**
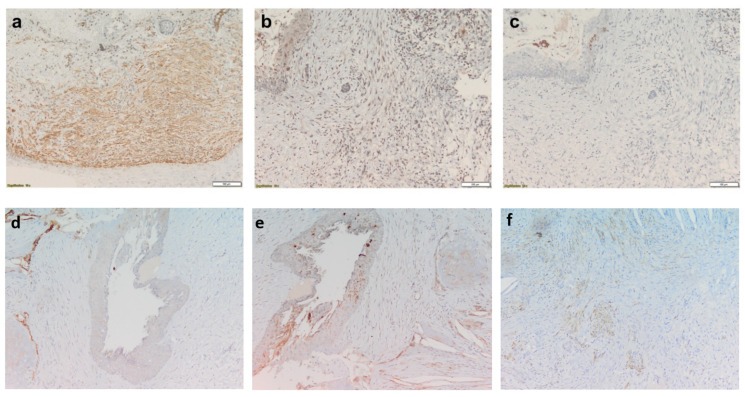
NBCCS-OKC, magnifications ×10. (**a**), αSMA markedly expressed in plump fibroblasts both in the epithelium and in the connective tissue, ×10; (**b**), MMP-7 moderately expressed in the MFs localized in the sub-epithelium and in the epithelial layer, ×10; (**c**), MMP-9 markedly detected in the endothelial cells and moderately in the sub-epithelial MFs, ×10; (**d**), Recurrent OKC, αSMA expression around the vessels, ×10; (**e**), Solitary OKC, MMP-7 expression in the inflammatory area and in the basal and intermediate connective wall, ×10; (**f**), Solitary OKC, MMP-9 slightly expressed in the basal-intermediate connective wall and moderately in the phlogosis areas, ×10.

**Figure 3:**
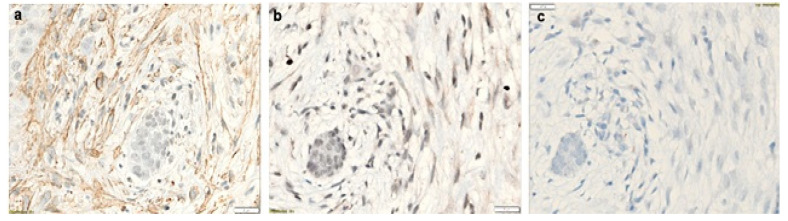
NBCCS-OKC (same case in Figure 2a–c), magnifications ×40. (**a**), αSMA expression, ×40; (**b**), MMP-7 expression, ×40; (**c**), MMP-9 expression, ×40.

In NBCCS-OKCs specimens, MMP-7 was observed in the MFs localized in the sub-epithelium and in the deep connective wall. Invading droplets showed high expression of MMP-7 in NBCCS-OKCs (Figure 1c). A moderate expression was detected in the epithelial layer ECM and in the endothelial cells (Figure 2b and Figure 3b). Furthermore, solitary OKCs showed a marked expression of MMP-7 in the inflammatory areas and in the basal and intermediate connective wall (Figure 2e), while recurrent OKCs showed a lower expression of MMP-7 in the epithelium, in the full thickness of the connective wall and in the phlogosis areas.

### 3.2. MMP-9 Expression

In the solitary OKCs specimens, MMP-9 was slightly expressed in the basal-intermediate connective wall and moderately in the phlogosis areas. The epithelium was negative for MMP-9, whereas in recurrent OKCs MMP-9 was markedly expressed in the basal-intermediate connective wall, especially in acantholytic areas. A moderate expression was also found in the epithelium and daughter cysts of recurrent OKCs. NBCCS-OKCs specimens showed a different MMP-9 expression pattern: it was markedly detected in the endothelial cells, moderately in the sub-epithelial MFs and in the deep connective wall and slightly in the epithelium (Figure 2c and Figure 3c). Invading droplets showed a lower level of expression of MMP-9 in NBCCS-OKCs compared to MMP-7 (Figure 1d).

### 3.3. Desmin Expression

**Figure 4:**
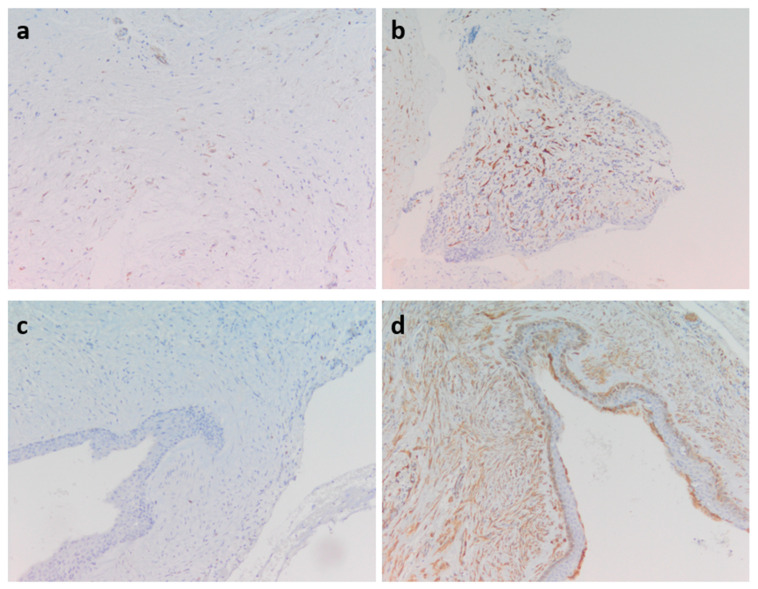
(**a**), NBCCS-OKC, moderate desmin expression around the vessels, ×10; (**b**), Solitary OKC, caldesmon scattered expression, ×10; (**c**), Sporadic OKC, focal desmin expression, ×10; (**d**), NBCCS-OKC, caldesmon in plump fibroblasts in the epithelium and throughout the connective wall, ×10.

Desmin showed a mild-moderate expression around the vessels of solitary OKCs specimens and a focal expression in sporadic OKCs (Figure 4c). We did not find desmin expression in most of the recurrent and syndromic OKCs specimens. However, few NBCCS-OKCs specimens showed a moderate desmin expression around the vessels (Figure 4a).

### 3.4. αSMA, Caldesmon and MF Expression

αSMA was markedly expressed in plump fibroblasts both in the epithelium and throughout the connective wall in NBCCS-OKCs specimens (Figure 1a,b, Figure 2a and Figure 3a). In solitary OKCs αSMA was moderately expressed in plump fibroblasts and showed a mild to moderate concentration around the vessels. Sporadic OKCs specimens were characterized by a marked αSMA expression in the MFs organized in clusters. Furthermore, αSMA showed high expression around the vessels of recurrent OKCs specimens (Figure 2d).

A marked caldesmon expression was demonstrated in plump fibroblasts in the epithelium and throughout the connective wall in the NBCCS-OKCs (Figure 4d). Solitary OKCs specimens showed a mild scattered expression (Figure 4b).

NBCSS-OKCs were characterized by a greater mean number of MFs (200 ± 2.5) localized below the epithelium and throughout the connective wall and in the surrounding epithelial droplets. Recurrent and sporadic OKCs showed a lower mean number of MFs (100 ± 2.5 for recurrent and 150 ± 2.5 for sporadic) confined below the epithelium. Instead, solitary OKCs MFs (mean number: 100 ± 2.5) were identified in the deep connective wall.

**Figure 5:**
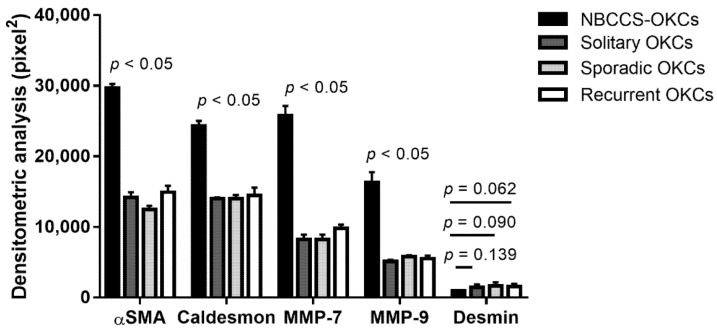
Densitometric analysis. A bar chart represents the comparison of the densitometric count (pixel2) of αSMA, caldesmon, MMP-7, MMP-9, and desmin immunostaining in the evaluated fields for NBCCS-OKCs tissue compared to solitary, sporadic and recurrent OKCs tissue, expressed by positive percentage, dark brown pixels of the analyzed fields. Data are presented as mean ± standard deviation (SD).

In Figure 5 the densitometric expression of MMP-7, MMP-9, desmin, αSMA, and caldesmon is shown. MMP-7, MMP-9, αSMA, and caldesmon were significantly increased in NBCCS-OKCs when compared to solitary, sporadic and recurrent OKC specimens (*p* < 0.05). Contrary, the variation in desmin immunoexpression between NBCCS-OKC vs. Solitary OKC data, NBCCS-OKC vs. Sporadic OKC data, and NBCCS-OKC vs. Recurrent OKC data were not statistically significant (*p* = 0.139, *p* = 0.090, and *p* = 0.062, respectively).

## 4. Discussion

The present study analyzed the immunohistochemical expression of MMP-7, MMP-9, αSMA, desmin and caldesmon in NBCCS-OKCs compared to recurrent and sporadic keratocysts.

OKCs show local aggressive behavior with a higher risk of recurrence than odontogenic cysts. These differences may be due to an increased epithelial mitotic activity in the suprabasal layers and by the enzymatic activity in OKC’s epithelial layer [27,28]. However, a new interest is emerging in studying the role of stromal tissue in the pathogenicity of OKC’s lesions and in the growth capacity of many neoplasms [29]. Even in many odontogenic lesions (such as odontogenic cysts and tumors) the presence of MF was reported [14]. Immunohistochemical studies showed that MF are distributed in the inner subepithelial layer of the cyst wall and in the outer region adjacent to the bone-facing surface, demonstrating that MF contributes to cyst wall elasticity limiting cyst expansion [14]. OKC’s and solid ameloblastomas showed an increased MFs’ mean number compared to dentigerous cysts, unicystic ameloblastomas, and ameloblastic fibroma, with values comparable to OSCC, suggesting that a higher amount of stromal MFs may be associated to the local aggressive behavior of these lesions [30]. However, other researchers did not find a significant difference in the MFs expression between sporadic OKCs and NBCCS-OKCs; therefore, these cells may not be related to the more aggressive biological behavior of NBCCS-OKCs [4]. However, according to Hong et al., fibroblasts derived from NBCCS-associated OKC showed higher proliferative and osteoclastogenic capacity than fibroblasts from other OKC variants [5]. Therefore, the different biological behavior may be explained not by the numerical difference but by the different metabolic activity of the fibroblasts in the OKC variants. This possibility could be validated by the results of the present study given the variety of expressions of caldesmon and αSMA and the different distribution of MF in the various types of OCKs.

Epithelial and mesenchymal cells release of MMPs could also explain the invasion mechanisms of OKCs [8]. In OKCs specimens, in general, we found that both parenchymal and stromal cells showed the expression of MMPs. This may explain the lithic processes in the ECM related to stromal cell activity. Some studies showed MMP-1 expression in the epithelial and connective layers of 80–100% of OKCs cases, but the higher expression of MMP-1 was observed in NBCCS-OKCs specimens [4]. MMP-2 expression in OKCs is higher than odontogenic cysts, which may explain the greater aggressiveness and tumor recurrence [31]; however, an investigation did not find a significant difference between the type of lesions [4]. MMP-7 is expressed in OKC and may promote a calcification process in these lesions [31]. Furthermore, MMP-7 appears to be able to trigger a cascade of activity of MMPs such as MMP-2 and MMP-9 to degrade many ECM components such as collagen type IV, elastin, and laminin. Confirming what was previously hypothesized, in the present study we observed a different pattern of MMP-7 expression in NBCCS-OCKs compared to solitary and recurrent OKCs.

Regarding MMP-9, different authors have observed its expression both in the epithelium (100% of OKCs specimens) and in the connective layer (85–100%) [4]. Similarly, we observed in OKC specimens the expression of MMP-9 in the connective tissue, endothelial cells and MFs with different expression patterns in NBCCS-OKCs. MMP-9 is more expressed in the connective tissue than pericoronal follicles, and according to de Andrade Santos et al., this may contribute to OKCs’ more aggressive behavior through the degradation of the ECM. Furthermore, MMP-9 was more markedly expressed in the OKCs’ epithelium compared to dentigerous cysts and radicular cysts [7].

This study is limited by the low number of patients. Furthermore, tissue from primary OKCs, before becoming a recurrent lesion, was not available. Therefore, we carried out this investigation in sporadic and recurrent OKCs which were not paired. However, findings from this research may prompt further studies in which will be useful to evaluate both and compare the paired findings.

## 5. Conclusions

The present study showed different expression patterns of MMP-7, MMP-9, α-SMA, desmin and caldesmon with a different distribution of MF in OKC specimens. However, further studies will be needed to evaluate whether the different expressions of these markers can be correlated to a different clinical behavior.

## Data Availability

The data that support the findings of this study are available from the corresponding author upon reasonable request.
